# Supplementary material for: Analysis and forecasts for trends of COVID-19 in Pakistan using Bayesian models
Source: PeerJ. 2021 Jul 7;9:e11537. doi: 10.7717/peerj.11537 (PMC8272466; doi:10.7717/peerj.11537)
Supplement: Supplemental Information 3 [file peerj-09-11537-s003.docx]

**Planner**

| Question | Hypothesis | Sampling plan | Analysis Plan | Interpretation |
| --- | --- | --- | --- | --- |
| Can we prefer the BSTS models (as compared to ARIMA models) for forecasting the patterns of COVID-19 in Pakistan |  |  | Comparison between forecast accuracy of BSTS models and ARIMA models using RMSE, MAE, RMSPE and RMdSPE as criteria of forecast accuracy | The results reported in Table 1, suggest that the values of RMSE, MAE, RMSPE and RMdSPE are smaller fro BSTS models as compared to ARIMA models. Hence the proposed models can be used more efficiently. |
| Are the assumptions of BSTS models fulfilled | The residual are white noise |  | For a parsimonious BSTS model, the residuals from the model should be white noise. We have tested this assumption using Ljung Box test | The results for the Ljung Box test, placed in Table 2, indicate that the residuals from the proposed models are white noise at different lags. Hence, the models are suitable for obtaining the said forecasts |
| Has the lifting of lockdown at the earlier stage increased the pace of the outbreak in the country |  |  | The causal impacts of lifting the lockdown have been investigated using intervention analysis under BSTS models. | The causal impacts of lifting the lockdown have been presented in Figure 2(B). The results elucidate the lifting of lockdown at the earlier stage has resulted into an absolute increase of 98,768 confirmed cases with 95% interval [85,544; 111,018] |
| What can be the future patterns of the pandemic in Pakistan |  |  | The forecasts for the patterns of the outbreak have been obtained using more flexible and more efficient BSTS models | on August 10, 2020, the expected number of positive cases in Pakistan will be 333,693 with 95% prediction interval [267,470; 393,702]. Similarly, the number of deaths in the country is expected to reach 7,178 [6,003; 8,368] and recoveries may grow to 265,619 [244,902; 290,985]. However, it was very encouraging to observe that the number of active cases is expected to decrease to 68,344 [22,568; 102,717] |
| How our study is different from the earlier contributions |  |  |  | This study aims to provide a more flexible analytical framework that decomposes the important components of the time series, incorporates the prior information, and captures the evolving nature of model parameters. This objective has been achieved by employing Bayesian structural time series (BSTS) models. |
